# Clinical Efficacy of *Cortex Daphnes (Zushima)* Patch in Patients With Symptomatic Knee Osteoarthritis: A Multicenter Non-Inferiority Randomized Controlled Clinical Trial

**DOI:** 10.3389/fphar.2021.646310

**Published:** 2021-05-07

**Authors:** Yan-Ting Li, Juan Jiao, Yi Zhang, Ci-Bo Huang, Hai-Dong Wang, Bei Wang, Xiao Su, Hui Song, Mian-Song Zhao, De-Xun Jiang, Jia-Qiang Wang, Quan Jiang

**Affiliations:** ^1^ College of Basic Medical Science, Zhejiang Chinese Medical University, Hangzhou, China; ^2^ Department of Rheumatology, Guang’anmen Hospital, China Academy of Chinese Medical Sciences, Beijing, China; ^3^ Department of Rheumatology, Beijing Hospital, Beijing, China; ^4^ Department of Rheumatology and Osteopathy, Gansu Provincial Hospital of TCM, Lanzhou, China; ^5^ Department of Rheumatology and Immunology, Beijing Hospital of Traditional Chinese Medicine, Capital Medical University, Beijing, China; ^6^ Department of Rheumatology, Shanghai Municipal Hospital of Traditional Chinese Medicine, Shanghai University of Traditional Chinese Medicine, Shanghai, China; ^7^ Department of Rheumatology and Immunology, Beijing Jishuitan Hospital, Beijing, China; ^8^ Department of Rheumatology and Clinical Immunology, Beijing Shijitan Hospital, Capital Medical University, Beijing, China; ^9^ Department of Rheumatism and Immunology, Seventh Medical Center of PLA General Hospital, Beijing, China; ^10^ Gansu Taikang Pharmaceutical Co., Ltd., Lan Zhou, China

**Keywords:** knee osteoarthritis, nonsteroidal anti-inflammatory drugs, Cortex Daphnes patch, pain, non-inferiority trial design

## Abstract

**Background:** Osteoarthritis (OA) is imposing substantial burdens on individuals and society with the aging population. Cortex Daphnes patch is widely used for symptomatic knee OA in China with a satisfying clinical efficacy; however, there is scant clinical evidence supporting its use. To evaluate its efficacy, we conducted a multicenter, non-inferiority, randomized, parallel-group study comparing Cortex Daphnes patch with topical nonsteroidal anti-inflammatory drugs in patients with knee OA (NCT02770950).

**Methods:** A total of 264 symptomatic knee OA patients were treated with Cortex Daphnes or indomethacin cataplasms applied to affected sites once daily for 2 weeks. The primary outcome was improvement in knee pain on walking as assessed using a visual analog scale (VAS). The non-inferiority margin based on the full analysis population was set as –5 mm on the pain VAS. The secondary outcomes were changes of the Western Ontario and McMaster Universities Osteoarthritis Index (WOMAC) total score, WOMAC scores for pain, function and stiffness, the 36-item Short Form Health Survey (SF-36), and global assessment of knees by the patients. Responder rates for pain VAS, WOMAC total score, and WOMAC pain were also included in the secondary outcomes.

**Results:** The Cortex Daphnes patch was non-inferior to indomethacin cataplasms for the primary outcome with a group difference (Cortex Daphnes patch–indomethacin cataplasm) of 2.1 mm (95% confidence interval: 2.1–6.4); similar results were found in the per-protocol population. For all other outcomes, no significant differences were found in the full analysis set or in the per-protocol analysis set, except the responder rates for WOMAC pain was higher in the Cortex Daphnes patch group than in the indomethacin cataplasm group (78.4 vs. 64.7%, p = 0.022) in the per-protocol analysis set. Overall, 28.8% patients in the Cortex Daphnes patch group and 9.8% in the indomethacin cataplasm group reported treatment-related adverse events, the vast majority of which were mild-to-moderate skin irritation, resulting in only 3.8 and 0.8% of patients dropping out, respectively.

**Conclusion:** The Cortex Daphnes patch, which provides satisfactory analgesic efficacy and enhances the physical function of the knee, as well as improving quality of life, may be a promising alternative to knee OA.

## Introduction

Osteoarthritis (OA) is a progressive disease of joints, common in middle and old age, that leads to joint capsule and ligament contracture, resulting in joint pain and functional impairment, as well as social and economic burdens (Dieppe and Lohmander, 2005; Hunter et al., 2014; Sharif et al., 2017). The joint pain, deformity, and motor dysfunction caused by OA can further increase the incidence of cardiovascular events and all-cause mortality (Xing et al., 2016; Liu et al., 2017). Knee OA is the most common type of OA; symptomatic knee OA is associated with considerable morbidity both in China and elsewhere (Cooper and Arden., 2011; Tang et al., 2016), with the result of an almost 90% increase in all-cause mortality (Liu et al., 2015). There is no specific treatment for osteoarthritis so far. Nonsteroidal anti-inflammatory drugs (NSAIDs) have been recognized as a good choice to manage symptomatic knee OA; however, oral NSAIDs are associated with higher risk of gastrointestinal, cardiovascular, and renal injury than placebo (Cepeda et al., 2006). Topical NSAIDs can avoid many of the adverse effects associated with systemic medications, can be considered as alternative therapy by patients with knee OA (Ringdahl and Pandit, 2011), but in fact, topical NSAIDs were less effective than oral NSAIDs (Lin et al., 2004). Therefore, it is of great significance to develop novel and more efficient therapeutic strategies for treatment of knee OA.

Traditional Chinese medicine has been accepted as a complementary therapy for knee OA, owing to its effects on relieving pain and improving functions of knee joint (Wang et al., 2020). Topical Chinese herbal preparation, as a representative form of traditional Chinese medicine, is commonly used in patients with symptomatic knee OA. Also known as Zushima (pronounced/zu-shi-ma:/), Cortex Daphnes is the processed stem and root barks of Daphne giraldii Nitsche., Daphne tangutica Maxim., and Daphne retusa Hemsl., all of which belong to the genus Daphne (Thymelaeaceae) (Pharmacopoeia of the People’s Republic of China, 1977).

According to Chinese medicine theory, the function of Cortex Daphnes herb is to remove blood stasis and relieve pain, removing cold-wind and dredge collaterals. It is used to treat headache, stomach ache, bruises, limb numbness, joint pain, and others. Although Cortex Daphnes patch has been widely used to treat arthritis in China, there have been few clinical studies reporting its effect and safety. The first published clinical study of Cortex Daphnes patch was in the 1980s, reporting the effects of pain relief in Chinese patients with soft tissue injury (Wang, 1986). Only one low-quality randomized controlled trial in patients with knee OA reported satisfactory clinical efficacy of Cortex Daphnes patch when combined with acupuncture (Wang and Zhan, 2014).

The aim of the present study is to evaluate whether Cortex Daphnes patch has the potential to be a valuable topical intervention for patients with knee OA. For this reason, we conducted a multicenter, non-inferiority, randomized controlled trial to compare the safety and efficacy of the Cortex Daphnes patch to indomethacin cataplasms for the treatment of knee OA.

## Materials and Methods

### Plant Material, Handling, and Phytochemical Analysis

Cortex Daphnes is majorly derived from the field-grown root bark and stem bark of Daphne giraldii Nitsche., which were collected in the plateau region of Gansu Province, China, and processed as described in the standard of Chinese medicinal materials in Gansu Province (Gansu Medical Products Administration, 2009). The main processing methods include rinsing, drying, and cutting pharmaceutical materials. Cortex Daphnes patch is the inheritance and innovation of traditional techniques of the black plaster. The black plaster, as a representative form of topical Chinese herbal preparation, has been used in China for at least thousands of years. The processing and extraction of Cortex Daphnes patch mainly include the following steps: Cortex Daphnes decoction pieces are cut off and boiled twice with water. Then, the decoction is filtered, concentrated, and dried. Finally, the Cortex Daphnes dry powder is added to the traditional black plaster matrix that is refined and smeared in the center of the plaster cloth. Cortex Daphnes patch is made by adding water-extracted Cortex Daphnes dry powder instead of the traditional frying extraction method, so as to avoid extreme damage to the active ingredients. Cortex Daphnes patch in the current study was produced by Gansu Taikang Pharmaceutical Co., Ltd. (China, batch number: 20150679). Preparation and assay methods of Cortex Daphnes patch are based on the pharmaceutical standards of the Ministry of Health of the People’s Republic of China (Standard No. WS3-B-3456-98; Pharmacopoeia Committee of National Health, 1998). Its phytochemical content (daphnetin) in each patch, according to extraction and assay methods, was 600 µg dry weight. The chemical profiling of Cortex Daphnes patch was detected using LC/MS/MS method, and the details were provided in supplementary material.

### Study Design

This was a multicenter, randomized, active controlled trial with duration of 2 weeks to evaluate the efficacy and safety/tolerability of Cortex Daphnes patch in patients with symptomatic knee OA. The study was conducted at eight sites in China from May 2016 to December 2017.

Among those NSAIDs used for local treatment of OA, topical administration of indomethacin has been widely prescribed for pain in China. According to package instructions, the dose of indomethacin cataplasm was one patch for one site once daily for 24 h.

### Selection Criteria

Participants were all outpatient patients who visited doctors complaining of knee pain. Patients were aged 40–75 years who met the 1995 American College of Rheumatology combined clinical and radiographic criteria for knee OA (Hochberg et al., 1995) and had symptoms in both knees. The inclusion criteria were knee pain of no less than 20 mm on a 100-mm visual analog scale (VAS) when walking flat. Patients taking oral NSAIDs prior to the breakthrough period (one week before starting) were included. The exclusion criteria included swollen and hot knees at the time of recruitment and patients with other joint diseases, such as rheumatoid arthritis, ankylosing spondylitis, congestive heart failure and edema, and advanced renal disease. We also excluded patients who were allergic to any ingredient in Cortex Daphnes patch (Cortex Daphnes and substrates including lithargite, linseed oil, and red lead) and in indomethacin cataplasm, as well as those who had punctured knee skin or other skin diseases.

### Randomization and Masking

This was a multicenter and active-controlled design. Using the block randomization method, a random number table was generated by an independent third party, the Clinical Evaluation Center of China Academy of Chinese Medical Sciences, using SAS 9.4 software (SAS Institute, Cary, NC, United States). The randomized assignment sequence was placed in a sealed opaque envelope and was kept by the scientific research management department of the research unit and can be reproduced upon request. Eligible patients were randomized 1:1 to either Cortex Daphnes or indomethacin cataplasm topical treatment. Prior to recruitment, study investigators, site staff, and patients were blinded to the details of the allocation sequence. In order to avoid patient’s preference for treatment method, we discussed all the interventions (the Cortex Daphnes patch and the indomethacin cataplasm) involved in this study with each participant, and informed them that all the treatments may benefit them. Furthermore, we asked a staff member not to discuss the intervention with participants when dispensing the medicine. The therapeutic and side effects were evaluated by a trained staff member of each site who was blinded to group allocation.

### Outcome Measures

The study included a screening visit to determine eligibility, a baseline visit, and a 2-week visit. Patient-reported outcome measures were collected at baseline and at the 2-week visit. Unscheduled visits were also possible at any time during the study treatment if required.

The primary outcome was change in the patient’s global assessment of pain intensity score from baseline to posttreatment. The Patient’s Global Assessment of Pain Intensity score employed a 100-mm VAS pain scale evaluating knee pain when walking flat (0 for no pain and 100 for pain as bad as it could possibly be) (Cao et al., 2009). The secondary outcomes included changes in the Western Ontario and McMaster Universities Osteoarthritis Index (WOMAC) total score, WOMAC scores for three subscales (pain, function, and stiffness), the 36-item Short Form Health Survey (SF-36), and the patient’s global assessment of knees from baseline to posttreatment. Responder rates for pain VAS (at least a 30% improvement from baseline), WOMAC total score (at least a 30% improvement from baseline), and WOMAC pain (at least a 30% improvement from baseline) were also included in the secondary outcomes. The WOMAC is a 24-item disease-specific patient-reported outcome measure with five questions assessing pain (range 0–20), two assessing stiffness (range 0–8), and 17 assessing function (range 0–68) (Bellamy et al., 1988). The WOMAC total score ranges from 0 to 96, with higher scores indicating greater burden of knee OA (Bellamy et al., 1988). The SF-36 is a generic quality of life instrument with eight health domains. It consists of 36 questions and assesses eight dimensions: physical functioning, role physical, pain index, general health, vitality, social functioning, role emotional, and mental health index (Li et al., 2002). It also provides two summary measures of physical and mental components, physical component score and mental component score, ranging from 0 to 100, with higher scores indicating better health status (Li et al., 2002). The participants were all self-assessed using VAS score to document the overall symptom severity of their knees at the beginning of the study and after treatment.

Safety evaluations included monitoring of adverse events (AEs), treatment discontinuations, measurement of skin irritation, and the assessment of clinical laboratory investigations. Skin irritation intensity evaluation criteria were used to record adverse skin events, which were the most commonly expected side effects. According to these criteria, the degree of the irritation symptoms including erythema, edema, and pruritus, were divided into mild, moderate, and strong irritation and were assessed by a trained physician (Bureau of Drug Policy and Administration of the People’s Republic of China, 1994). The specific laboratory assessments included complete blood counts and urinalyses, as well as measurements of glutamic-pyruvic transaminase, glutamic-oxalacetic transaminase, creatinine, and blood urea nitrogen.

### Treatment

**FIGURE 1:**
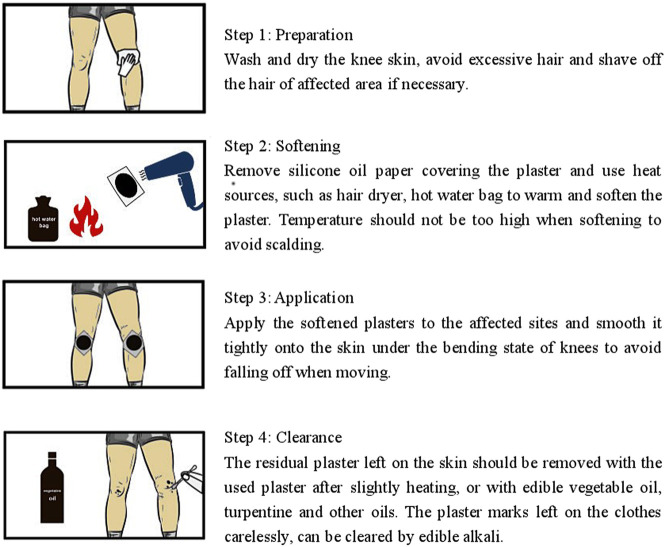
Application of Cortex Daphnes plaster.

Patients whose eligibility was confirmed at the baseline visit were randomly assigned to treatment with one or the other intervention. During the treatment period, participants were instructed to apply the Cortex Daphnes patch or the indomethacin cataplasm (Nipro Patch CO. LTD. Japan, batch number: EM002) onto clean skin overlying the affected sites on both knees every night and keep it in place for 24 h. The standard operation procedure for the application of Cortex Daphnes patch has been established. As shown in Figure 1, instructions were offered to every participant in the Cortex Daphnes patch group to standardize the external use site, the administration time, and the method of placing and removing the patch to ensure the consistency of application.

No other treatments for knee OA were permitted during the treatment period: systemic and topical NSAIDs; opioid analgesics; acetaminophen; oral and intra-articular injection of corticosteroids; intra-articular knee injections (hyaluronic acid injection and local anesthetics); surgical or physiotherapeutic therapies; acupuncture; and aspirin. All AEs were recorded in detail and were monitored regularly until properly resolved or the condition was stable.

### Sample Size Calculation

The sample size was determined using the primary endpoint of the change of VAS pain score. The literature showed that the effect for Cortex Daphnes patch with the same primary endpoint was 44.6 mm (SD: 33.4 mm) and it was 35.3 mm (SD: 30.4 mm) for indomethacin cataplasms at week 2 (Wang et al., 2016). Assuming a non-inferiority margin of -5 mm and that the ratio of the Cortex Daphnes patch group and the indomethacin cataplasm group was 1:1, using a two-sided test with a significance level (α) of 0.05 and a power (1–β) of 0.90, the required sample size was estimated at 105 cases in each group. Allowing for a dropout of 20%, the Cortex Daphnes patch group and the indomethacin cataplasm group each required 132 patients, a total of 264 patients.

### Statistical Analysis of Data

Statistical analyses were performed using SAS version 9.4 (SAS Institute, Cary, NC, USA). The full analysis set (FAS) and per-protocol (PP) analysis set were analyzed. The FAS population included all participants who were randomized and underwent the assigned treatment. Non-inferiority of the primary endpoint (change of VAS pain score of Cortex Daphnes patch vs. indomethacin cataplasm) was determined if the lower limit of the 95% CI for the difference was not less than the cutoff value of -5 mm. The changes of primary and secondary endpoints at week 2 from baseline were analyzed using an analysis of covariance (ANCOVA) model with the treatment group and the study center as factors and the baseline score as a covariate. The point estimate, the least squares (LS) means, and the two-sided 95% CI based on the ANCOVA model were provided for the difference between treatment groups. The 30% responders were defined as patients who experienced a 30% reduction of WOMAC pain score, WOMAC total score, and VAS score from baseline for each respective outcome. A p-value of <0.05 was considered statistically significant. All patients who received at least one dose of the study drug were included in the safety set (SS). Incidence of AEs, treatment-related AEs, and discontinuation rate due to AEs were compared between the two groups using the Pearson chi-square test and Fisher’s exact model.

## Results

### Baseline of Clinical Cohorts

**FIGURE 2:**
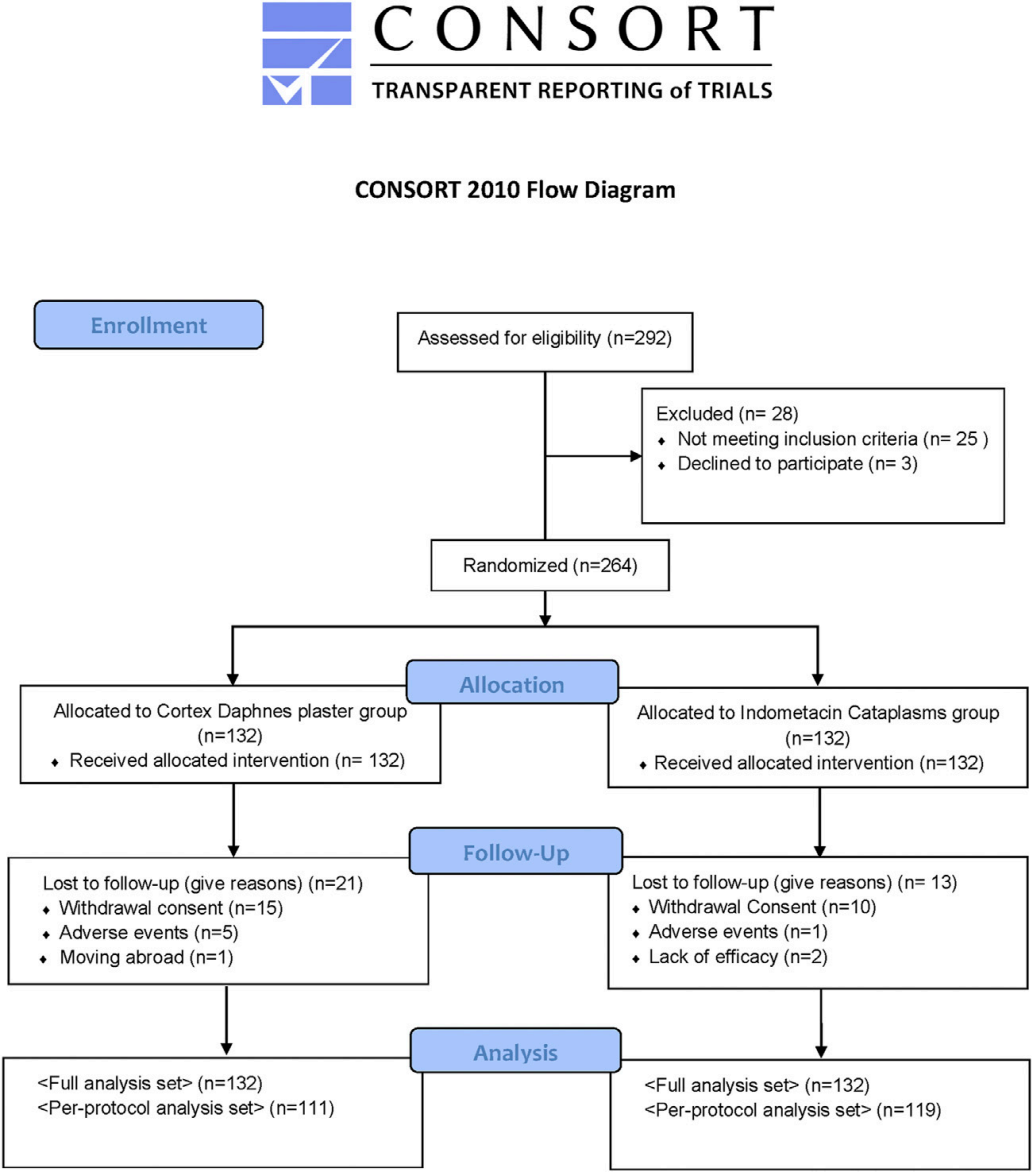
Flow diagram of the trial.

All patients were Chinese. Among 292 patients with symptomatic knee OA who were initially screened, 264 eligible patients were enrolled and constituted the FAS population. There were no statistically significant differences between the groups with respect to demographic and baseline clinical characteristics (Table 1). There were 21 (15.9%) patients in the Cortex Daphnes patch group and 13 (9.8%) patients in the indomethacin cataplasm group who did not complete the 2-week treatment, resulting in an overall dropout rate of 12.9% (Figure 2). Finally, 230 patients were included in the PP population for the efficacy analyses.

**TABLE 1:** Demographic and baseline clinical characteristics of patients in FAS population.

Variable	Cortex Daphnes patch group (*n* = 132)	Indomethacin cataplasm group (*n* = 132)	*p*-value
Age (years, mean (SD))	59.8 (7.8)	59.5 (8.6)	0.81
Gender (n (%))			0.15
Male	19 (14.4)	28 (21.2)	
Female	113 (85.6)	104 (78.8)	
Race (n (%))			0.31
Han	129 (97.7)	131 (99.2)	
Non-Han	3 (2.3)	1 (0.8)	
Duration of knee pain (month, median (Qd))	36 (48.0)	36 (52.5)	0.93
Weight (kg, mean (SD))	64.1 (8.8)	66.1 (11.7)	0.13
Height (cm, mean (SD))	162.1 (6.5)	161.9 (5.8)	0.83
BMI (kg/m^2^, mean (SD))	24.4 (3.0)	25.2 (4.1)	0.08
Kellgren/Lawrence scale (n (%))			0.79
Grade 1	37 (28.0)	42 (31.8)	
Grade 2	60 (45.5)	45 (34.1)	
Grade 3	22 (16.7)	33 (25.0)	
Grade 4	1 (0.8)	0 (0)	
Unknown	12 (9.1)	12 (9.1)	
Pain VAS^a^ (mean (SD))	63.1 (14.0)	64.0 (16.1)	0.48
NRS^b^ (n (%))			0.30
NRS-mild pain	1 (0.8)	5 (3.8)	
NRS-moderate pain	78 (59.1)	63 (67.7)	
NRS-severe pain	53 (40.2)	64 (48.5)	
Patient’s global assessment of knee OA (mean (SD))	62.0 (14.3)	63.5 (16.5)	0.34
WOMAC^c^ (mean (SD))			
Total score	31.5 (15.2)	33.3 (15.5)	0.25
Pain	6.7 (3.2)	7.4 (3.6)	0.09
Stiffness	2.6 (1.8)	2.7 (1.8)	0.53
Function	22.3 (11.4)	23.2 (11.5)	0.42
SF-36^d^ (mean (SD))			
PCS	53.11 (8.8)	49.9 (19.7)	0.16
MCS	59.3 (18.3)	55.1 (22.6)	0.33

Abbreviations: FAS = full analysis set; BMI = body mass index; pain VAS = pain visual analog scale; NRS = numerical rating scale for pain; OA = osteoarthritis. WOMAC = Western Ontario and McMaster Universities Osteoarthritis Index; SF-36 = Short Form-36 Health Status Questionnaire; PCS = physical component score; MCS = mental component score.

a Pain VAS is a measurement for body pain. Scores range from 0 to 100 mm, with higher score indicating greater pain.

b The NRS allows a person to describe the intensity of his/her pain, as a number ranging from 0 to 10. “0” indicates “no pain,” “1 to 3” “mild pain,” “4 to 6” “moderate pain,” “7 to 9” “severe pain,” and “10” “bad as it could be.”

c The WOMAC is a 24-item disease-specific outcome measure and the higher score indicates greater burden of knee OA. Scores range from 0 to 96 with five questions assessing pain (range 0–20), two assessing stiffness (range 0–8), and 17 assessing function (range 0–68).

d SF-36 is a self-administered, 36-item questionnaire that assesses the physical and mental quality of life. Both of physical and mental component summaries can be combined ranging from 0 to 100, with higher scores indicating better health status.

### Treatment Effects

#### The Primary Outcome: Patient’s Global Assessment of Pain Intensity

In the FAS population, the LS mean changes from baseline in the pain VAS score at week 2 was –25.4 mm in patients receiving the Cortex Daphnes patch and –23.3 mm in patients receiving indomethacin cataplasms. The treatment difference (Cortex Daphnes patch–indomethacin cataplasm) in the FAS population was 2.1 mm and the two-sided 95% CI for the treatment difference was –2.1–6.4 mm. In the PP population, the result was similar (treatment difference and two-sided 95% CI: 3.8 [–0.6 to 8.2] mm). The lower limit of the 95% CI was –5 mm, which indicates that Cortex Daphnes patch was non-inferior to indomethacin cataplasm for the treatment of Chinese patients with knee OA.

### Secondary Outcomes

In the FAS population, there were no significant differences between groups with respect to any secondary endpoint after 2-week treatment (Table 2). The LS mean change from baseline in WOMAC total score was –13.9 in the Cortex Daphnes patch group and it was –12.6 in the indomethacin cataplasm group. WOMAC pain, stiffness, and function subscales were –3.1, –1.2, and –9.7 in the Cortex Daphnes patch group and –2.7, –1.1, and –8.9 in the indomethacin cataplasm group, respectively. The LS mean changes from baseline in SF-36 physical component scores and mental component scores were 9.3 and 5.8 in the Cortex Daphnes patch group and 8.7 and 5.2 in the indomethacin cataplasm group, respectively. The LS mean change from baseline in patient’s global assessment of disease activity was –24.6 in the Cortex Daphnes patch group and –23.2 in the indomethacin cataplasm group. In the PP population, the treatment differences between the groups for all secondary endpoints were similar (Table 3).

**TABLE 2:** Outcomes changes from baseline and difference in LS mean change of Cortex Daphnes patch from indomethacin cataplasms in FAS population posttreatment.

Outcomes	LS mean (95% CI) change from baseline		Between-group difference	*p-*value
	Cortex Daphnes patch group (n = 132)	Indomethacin cataplasm group (n = 132)	LS mean difference from control (95% CI)	
Pain VAS^a^	–25.4 (–28.4, –22.4)	–23.3 (–26.3, –20.3)	2.1 (–2.1, 6.4)	0.33
Patient’s global assessment	–24.6 (–27.6, –21.6)	–23.2 (–26.2, –20.3)	1.4 (–2.8, 5.6)	0.52
WOMAC^b^				
Total score	–13.9 (–15.7, –12.2)	–12.6 (–14.4, –10.8)	1.3 (–1.2, 3.8)	0.29
Pain	–3.1 (–3.5, –2.7)	–2.7 (–3.1, –2.3)	0.4 (–0.1, 1.0)	0.12
Stiffness	–1.2 (–1.4, –1.0)	–1.1 (–1.3, –0.9)	0.1 (–0.2, 0.4)	0.41
Function	–9.7 (–11.0, –8.3)	–8.9 (–10.2, –7.5)	0.8 (–1.1, 2.7)	0.41
SF-36^c^				
PCS	9.3 (7.2, 11.4)	8.7 (6.6, 10.8)	–0.6 (–3.6, 2.4)	0.70
MCS	5.8 (4.0, 7.8)	5.2 (3.3, 7.2)	–0.6 (–3.4, 2.1)	0.66

All values are means (95% confidence intervals). Abbreviations: FAS = full analysis set; WOMAC = Western Ontario and McMaster Universities Osteoarthritis Index; SF-36 = Short Form-36 Health Status Questionnaire; PCS = physical component score; MCS = mental component score.

a Pain VAS is a measurement for body pain. Scores range from 0 to 100 mm, with higher scores indicating greater pain.

b The WOMAC is a 24-item disease-specific outcome measure and the higher score indicates greater burden of knee OA. Scores range from 0 to 96 with five questions assessing pain (range 0–20), two assessing stiffness (range 0–8), and 17 assessing function (range 0–68).

c SF-36 is a self-administered, 36-item questionnaire that assesses the physical and mental quality of life. Both of physical and mental component summaries can be combined ranging from 0 to 100, with higher scores indicating better health status.

**TABLE 3:** Outcomes changes from baseline and difference in LS mean change of Cortex Daphnes patch from indomethacin cataplasms in PP population posttreatment.

Outcomes	LS mean (95% CI) change from baseline		Between-group difference	p*-*value
	Cortex Daphnes patch group (n = 111)	Indomethacin cataplasm group (n = 119)	LS mean difference from control (95% CI)	
Pain VAS^a^	–29.4 (–32.6, –26.3)	–25.6 (–28.6, –22.6)	3.8 (–0.6, 8.2)	0.09
Patient’s global assessment	–28.6 (–31.7, –25.5)	–25.5 (–28.5, –22.5)	3.1 (–1.2, 7.4)	0.16
WOMAC^b^				
Total score	–16.0 (–17.9, –14.2)	–13.8 (–15.6, –12.1)	2.2 (–0.4, 4.7)	0.10
Pain	–3.5 (–3.9, –3.1)	–2.9 (–3.4, –2.5)	0.6 (–0.0, 1.2)	0.06
Stiffness	–1.3 (–1.5, –1.1)	–1.2 (–1.4, –1.0)	0.1 (–0.2, 0.4)	0.39
Function	–11.2 (–12.6, –9.8)	–9.7 (–11.1, –8.4)	1.5 (–0.5, 3.4)	0.14
09SF-36^c^				
PCS	11.1 (8.7, 13.5)	10.0 (7.7, 12.4)	–1.1 (–4.4, 2.3)	0.53
MCS	7.1 (4.9, 9.4)	5.9 (3.7, 8.1)	–1.2 (–4.3, 1.9)	0.45

All values are means (95% confidence intervals).

Abbreviations: FAS = full analysis set; WOMAC = Western Ontario and McMaster Universities Osteoarthritis Index; SF-36 = Short Form-36 Health Status Questionnaire; PCS = physical component score; MCS = mental component score.

a Pain VAS is a measurement for body pain. Scores range from 0 to 100 mm, with higher scores indicating greater pain.

b The WOMAC is a 24-item disease-specific outcome measure and the higher score indicates greater burden of knee OA. Scores range from 0 to 96 with 5 questions assessing pain (range 0–20), 2 assessing stiffness (range 0–8), and 17 assessing function (range 0–68).

c SF-36 is a self-administered, 36-item questionnaire that assesses the physical and mental quality of life. Both of physical and mental component summaries can be combined ranging from 0 to 100, with higher scores indicating better health status.

**FIGURE 3:**
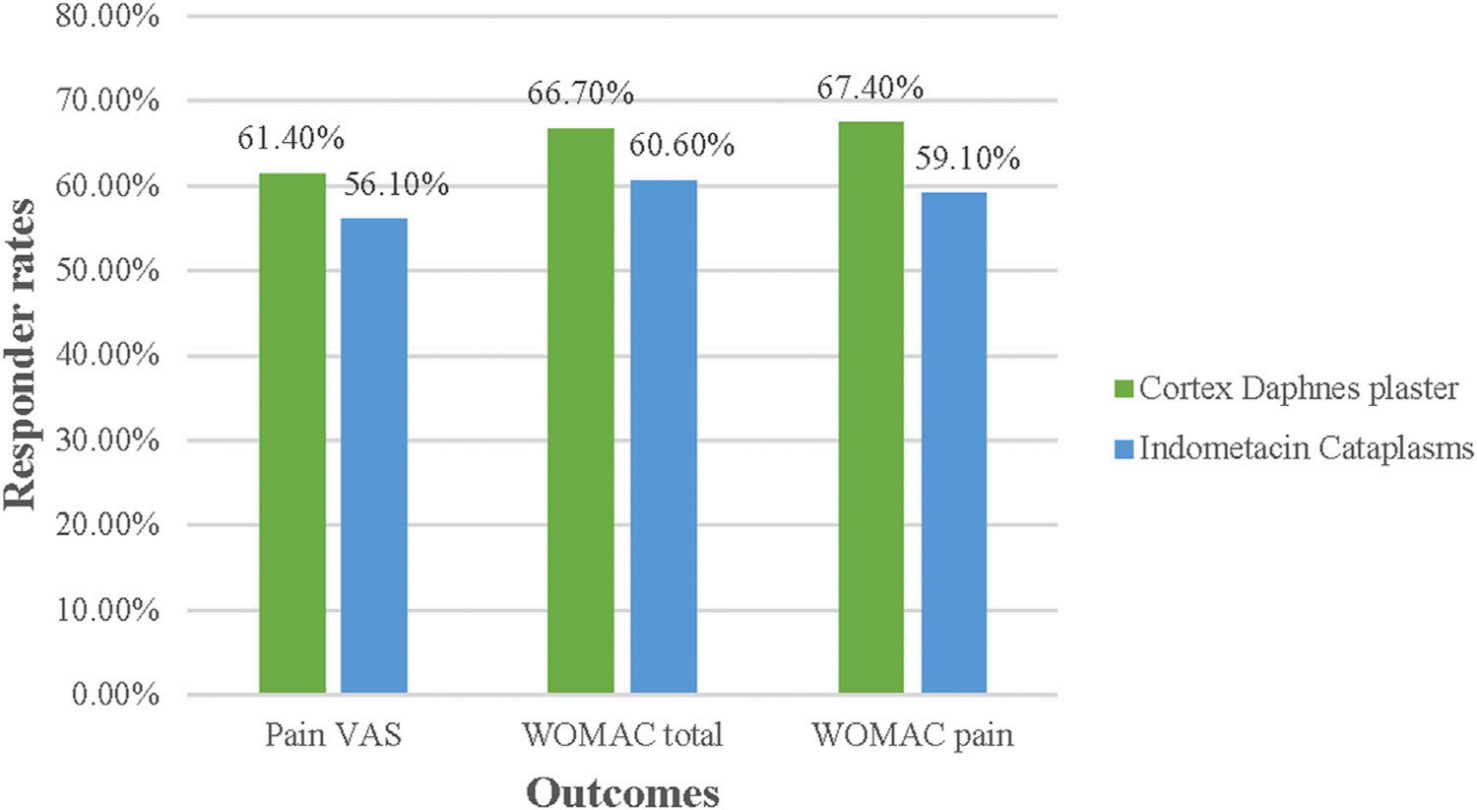
Responder rates for Pain VAS, WOMAC total, and pain score in FAS population posttreatment.

**FIGURE 4:**
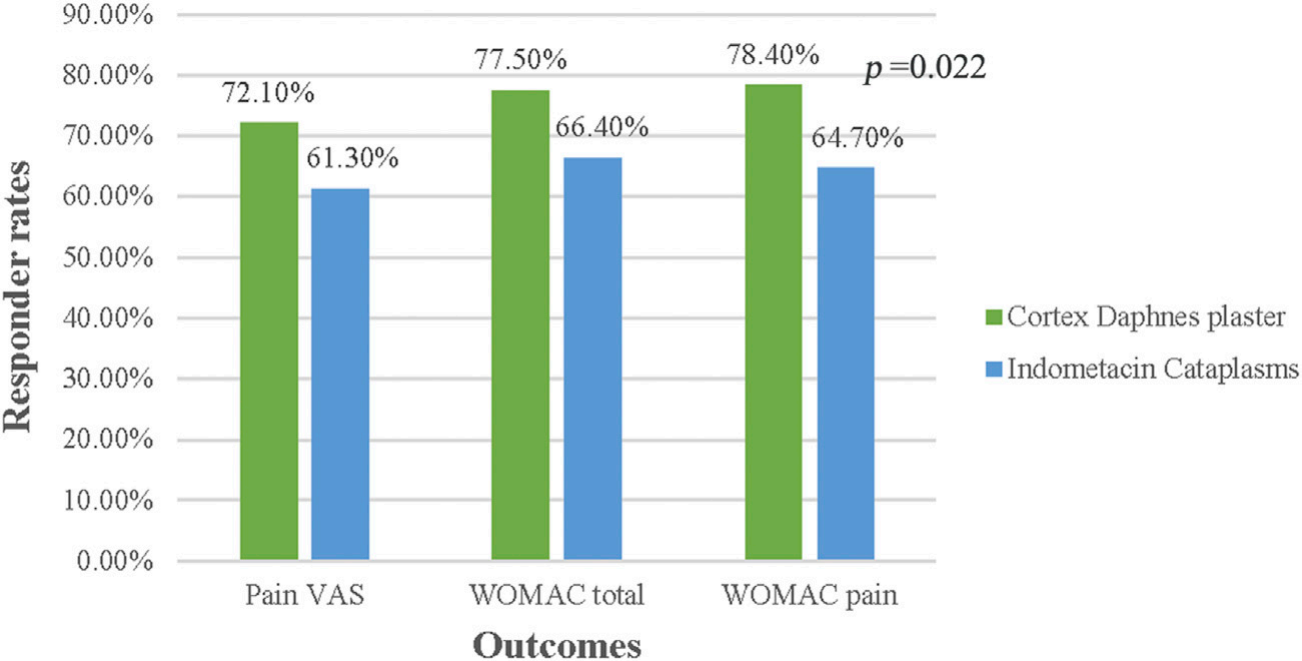
Responder rates for Pain VAS, WOMAC total, and pain score in PP population.

In the FAS population, no differences between groups were significant in terms of responder rates for pain VAS, WOMAC total score, and pain subscale (p > 0.05) (Figure 3). In the PP population, no significant differences were found, except that the percentage of 30% responder WOMAC pain subsacle was higher in the Cortex Daphnes patch group than in the indomethacin cataplasm group (78.4 vs. 64.7%, p = 0.022; Figure 4).

### Safety Profile

AEs are displayed in Table 4. AEs were documented 60 times during the study with a higher rate in the Cortex Daphnes patch group than in the indomethacin cataplasm group (29.5 vs. 19.5%, p = 0.008). The majority of these 60 events (51, 85%) were skin irritation and all were treatment-related AEs reported by 38 patients in the Cortex Daphnes patch group and 13 patients in the indomethacin cataplasm group. Routine laboratory examinations indicated that no patients in either group developed abnormalities. Most of the skin irritation AEs were mild-to-moderate in intensity with only five patients in the Cortex Daphnes patch group and one patient in the indomethacin cataplasm group discontinuing treatment.

**TABLE 4:** Number of patients experiencing adverse events during treatment.

Adverse effect	Cortex Daphnes patch group (*n* = 132)	Indomethacin cataplasm group (*n* = 132)	p*-*value
With AEs (n (%))	39 (29.5)	21 (15.9)	0.008
Skin irritation	38 (28.8)	13 (9.8)	<0.001
Sinus bradycardia	0 (0.0)	3 (2.3)	0.12
Upper respiratory infection	0 (0.0)	3 (2.3)	0.12
Gastrointestinal reaction	1 (0.8)	0 (0.0)	1.00
Thrombocytopenia	0 (0.0)	1 (0.8)	1.00
Pharyngitis	0 (0.0)	1 (0.8)	1.00
Treatment-related AEs (n (%))			
Skin irritation	38 (28.8)	13 (9.8)	<0.001
Discontinued due to AEs (n (%))			
Skin irritation	5 (3.8)	1 (0.8)	0.21

Abbreviations: AEs = adverse events.

The severity for skin AEs of erythema, itching, and edema are summarized in Table 5. Most expected side effects were slight in severity and resolved within 7 days without any additional intervention. The occurrence and severity of skin irritation, including erythema, itching, and edema tended to be higher in the Cortex Daphnes patch group than in the indomethacin cataplasm group. However, there were 38 patients in the Cortex Daphnes patch group reporting skin irritation symptoms. Among them, the knee skins of only 4 patients were allergic to the black plaster, while the other 34 patients reported that the knee skin around the plaster was allergic due to the mount material used in the Cortex Daphnes patch. Erythema and itching skin AEs resulting from black plaster were less likely to occur when using indomethacin cataplasms. There was one severe erythema and one itching skin AE in the Cortex Daphnes patch group due to black plaster, while no severe skin AEs occurred due to indomethacin cataplasms.

**TABLE 5:** The severity for the reported skin irritation.

Skin irritation	Cortex Daphnes patch group (*n* = 38)		Indomethacin cataplasm group (*n* = 13)
	Due to mount material (*n* = 34)	Due to black plaster (*n* = 4)	
Erythema			
Mild	9 (23.7)	0 (0.0)	6 (4.5)
Moderate	17 (44.7)	2 (5.2)	2 (1.5)
Severe	3 (7.9)	1 (2.6)	0 (0.0)
In total	29 (76.3)	3 (7.9)	8 (61.5)
Itching			
Mild	21 (55.3)	1 (2.6)	7 (5.3)
Moderate	8 (21.1)	1 (2.6)	1 (0.8)
Severe	1 (2.6)	1 (2.6)	0 (0.0)
In total	30 (78.9)	3 (7.9)	8 (61.5)
Edema			
Mild	5 (13.2)	1 (2.6)	1 (0.8)
Moderate	3 (7.9)	1 (2.6)	1 (0.8)
Severe	3 (7.9)	0 (0.0)	0 (0.0)
In total	11 (28.9)	2 (5.2)	2 (1.6)

## Discussion

Current international and Chinese guidelines support the use of topical NSAIDs as an early treatment option for symptomatic relief of the management of knee OA (Hochberg et al., 2012; McAlindon et al., 2014; Joint Surgery Group of Chinese Orthopedic Association, 2018). Despite extensive clinical applications, definitive evidence of the efficacy of the Cortex Daphnes patch as an alternate option has yet to be provided. Against this background, the current multicenter, randomized clinical trial showed that the Cortex Daphnes patch was not inferior to indomethacin cataplasms with respect to efficacy, and its safety profile was also favorable, with no serious AEs being observed. These observations strongly support the existing evidence to the effect that Cortex Daphnes patch may be a potential alternative topical application for treatment of knee OA.

Our results suggest that the Cortex Daphnes patch can effectively relieve the symptoms of knee OA, in line with the findings of previous clinical studies. Cui et al. demonstrated that a 2-week regimen of Cortex Daphnes patch could relieve pain, swelling, and restricted movement in patients with knee synovitis when compared with infrared radiation therapy (Cui and Liu, 2017). A randomized controlled clinical trial with a short duration of about 2 weeks reported that, when combined with acupuncture, Cortex Daphnes patch was superior to glucosamine sulfate in terms of pain relief and joint function improvement in patients with knee OA (Wang and Zhan, 2014). Due to its warm property, Cortex Daphnes patch even reduced wrist joint pain, swelling, and stiffness in cold-type patients with rheumatoid arthritis (Wang et al., 2016), as well as alleviating lumbar pain and promoting functional status when combined with herb decoction in cold-type patients with chronic lumbar muscle strains (Wu et al., 2020). The key differences between the current and previous studies involve differences in use of the Cortex Daphnes patch as well as different target patient populations. In our study, we used a single Cortex Daphnes patch in patients suffering from knee OA; the previous studies used single Cortex Daphnes patches in patients with other chronic pain or arthritis diseases, and in the only study of knee OA study, the intervention was Cortex Daphnes patch combined with acupuncture. Using a higher quality of design and well-recognized outcome measures, the current study is the first clinical evidence to suggest the effectiveness of Cortex Daphnes patch for treatment of symptomatic knee OA.

Our findings show that Cortex Daphnes patch has a favorable anti-inflammatory analgesic effect. In recent years, daphnetin, coumarins, lignins, flavonoids, diterpenes, and other chemical components have been isolated and identified from Cortex Daphnes. Daphnetin, the main phytochemical component of Cortex Daphnes, has provided dramatic improvement in percutaneous absorption results *in vivo* and *in vitro* experiments. Pharmacological activity experiments showed that prostaglandin E2 generation was inhibited by extract of Cortex Daphnes by inhibiting the expression of cyclooxygenase-2 (Yuan et al., 2009). An animal experiment showed that daphnetin significantly reduced the swelling of feet and the severity of arthritis in adjuvant-induced arthritis rat model (Gao et al., 2008). Daphnetin, a natural product, inhibits not only lipoxygenase and cyclooxygenase but also neutrophil-dependent superoxide anion generation (Fylaktakidou et al., 2004).

In recent years, more than 250 chemical constituents have been identified from the genus Daphne. The most important classes of compounds obtained from this genus include coumarins, flavonoids, lignans, terpenoids, and several other less common groups (Xu et al., 2011; Moshiashvili et al., 2020) Coumarins are among the most common compounds in the Daphne genus and major toxic components. Daphne genus–related potential risks include hepatotoxicity, nephrotoxicity, burning in the pharynx, gastrointestinal discomfort (nausea, vomiting, burning of stomach, gastroenteritis, and diarrhea), internal bleeding, muscle spasms and paralysis, and skin irritation (redness, swelling, and blusters). In severe cases, circulatory arrest and coma may be observed “(Goel et al., 2007; Xu et al., 2011; Moshiashvili et al., 2020; Wink, 2009; Stefanachi et al., 2018).” Therefore, we should pay more attention to the safety of the Cortex Daphnes patch. In the study, the safety profiles suggested a trend toward higher frequency and more severity of treatment-related adverse effects in the Cortex Daphnes patch group. Although skin irritation was the most frequently reported treatment-related adverse effect in both treatment groups, the material used in the Cortex Daphnes patch predisposes patients to increased risk of developing erythema and edema, as well as itching of the contact skin, especially after fixing the plasters for 24 h daily, for 2 weeks, on knee skin.

Fortunately, those skin side effects were mostly mild in severity, were transient, and did not require intervention. Moreover, the skin reactions may be alleviated after discontinuation of the Cortex Daphnes patches. These findings suggest a rest time for skin is needed when using the Cortex Daphnes patch, and the components containing the Cortex Daphnes patch should be changed to be milder and less irritating.

There are some limitations with respect to the design of this non-inferiority study. First, the completely different physical characteristics and smell between the Cortex Daphnes patch and the indomethacin cataplasm prevented double-dummy design from being applied. Nevertheless, considering that those two medicines and their topical simulators could not be used on the knee simultaneously, or even used separately on each knee, an enormous difference in efficacy might result in a substantial decline in compliance with the treatment plan. Therefore, to ensure the reliability of evaluation measurements, instead of using the double-dummy design, a staff member who dispensed the investigational medicine was trained not to discuss the intervention with participants, and the therapeutic effects and side effects were evaluated by another blinded trained staff member. Second, its long-term efficacy remains unclear due to a paucity of follow-up. Third, we did not pay attention on the average time for skin irritation after medication and relevant data need to be supplemented in the following Cortex Daphnes patch-related research.

## Conclusion

The Cortex Daphnes patch exerts favorable effects in decreasing knee pain and improving joint function for patients with knee OA, especially with mild-to-moderate knee OA, which were non-inferior to the therapeutic effects of a recognized treatment for knee OA. The clinical evidence presented here highlights the promising therapeutic efficacy of the Cortex Daphnes patch for the treatment of pain and symptoms associated with knee OA, particularly as part of a changing healthcare landscape that seeks to identify effective and safe plant medicines to treat chronic pain.

## Data Availability

The raw data supporting the conclusion of this article will be made available by the authors, without undue reservation.
