# Supplementary material for: Clinical Efficacy of Cortex Daphnes (Zushima) Patch in Patients With Symptomatic Knee Osteoarthritis: A Multicenter Non-Inferiority Randomized Controlled Clinical Trial
Source: Front Pharmacol. 2021 May 7;12:646310. doi: 10.3389/fphar.2021.646310 (PMC8137983; doi:10.3389/fphar.2021.646310)
Supplement: Supplementary file 1 [file DataSheet1.docx]

Supplementary Material

**Phytochemical Characterization of Cortex Daphnes patch**

# 1. Method: LC/MS/MS method

# 2. Sample preparation

The powder of Daphne giraldii were diluted 100-fold with ethanol and vortex-mixed. Following centrifugation the supernatant was transferred to a Waters Maximum Recovery vial and triplicate injections were analyzed.

# 3. LC conditions

system: ACQUITY UPLC (binary solvent manager, sample manager, HT column oven),

Column: Luna C18（φ4.6×250mm，5μm）

Column temperature: 35 °C

Injection volume: 5 µL

Flow rate: 1 mL/min

Mobile phase A: Acetonitrile

Mobile phase B: Water + 0.1% formic acid

| Time | Mobile phase A | Mobile phase B |
| --- | --- | --- |
| 0 | 18 | 82 |
| 20 | 18 | 82 |
| 35 | 33 | 67 |
| 50 | 33 | 67 |
| 60 | 18 | 82 |

**Table 1.** The gradient of Daphne giraldii

**4.** **MS conditions**

MS system: Acquity Xevo TQ-S (Waters, USA),

Ionization: ESI positive,

Capillary voltage: 5.5 kV,

Cone voltage: 10 V,

Source temperature: 150 °C,

Desolvation gas temperature: 600 °C,

Desolvation gas pressure: 60psi,

Desolvation gas: 800 L/hr,

Cone gas flow: 20 L/hr,

Collision gas: Argon.


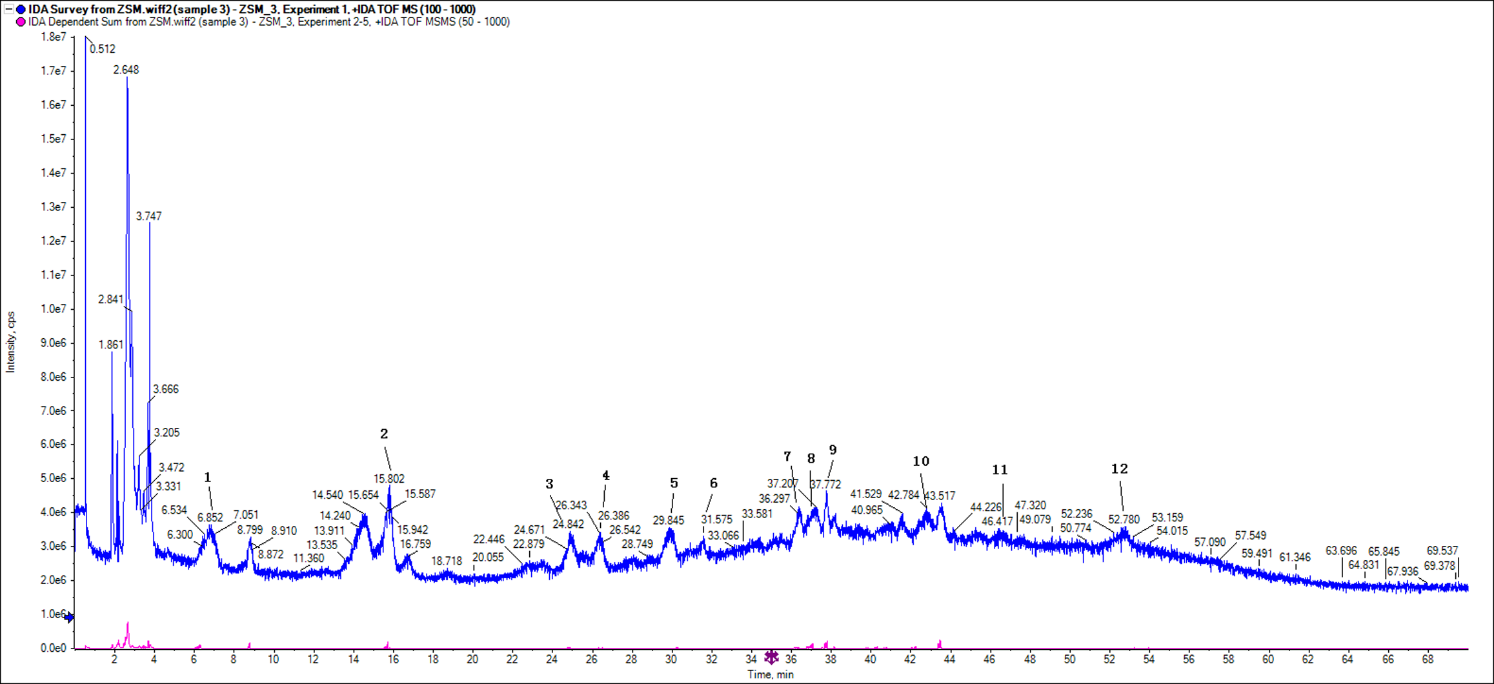


**Figure 1.** LC/MS/MS Chromatograms of representative compounds in Cortex Daphnes patch

**Table2.** Chemical profiling of Cortex Daphnes patch

| No. | Retention time (min) | Compound |
| --- | --- | --- |
| 1 | 6.852 | Daphnetin 7,8-di-o-β-D-glucoside |
| 2 | 15.802 | Daphnetin-8-o-β-D-glucoside |
| 3 | 24.842 | 8-hydroxy-7-methoxycoumarin |
| 4 | 26.386 | Daphnin |
| 5 | 29.845 | Daphnetin |
| 6 | 31.575 | 5-hydroxy-7-methoxyocumarin-8-O-β-D-gluocpyrnaoside |
| 7 | 36.297 | Syringoside |
| 8 | 37.207 | Umbelliferone |
| 9 | 37.772 | daphnoretin |
| 10 | 42.784 | woonenoside XI |
| 11 | 46.417 | oleanolic acid |
| 12 | 52.780 | Daphne flavane E/F |

Previous phytochemical studies of D. giraldii have identified more than 150 compounds including coumarins, flavnoids, lignans, diterpenes and miscellaneous ingredients, among which coumarins and flavonoids are the two major classes (Sun et al., 2008; Zhao et al., 2012; Sun et al., 2016, 2017). The content of total coumarins in D. giraldii was analyzed at the range of 18.44–31.73 mg/g, which showed a high similarity to that of D. tangutica from Shaanxi province (Su et al., 2009).

Reference

Wang, Q.M., Ni, Y.C., Xu, Y.Q., Ha, S.H., Cai, Y., 2000. The schizontocidal activity of daphnetin against malaria parasites in vitro and in vivo. Chin. J. Parasitol. Parasit. Dis.18, 204–206.

Sun, J., Wu, Z.J., Shen, Y.H., Zhang, C., Zhang, W.D., 2008. Studies on the chemical constituents of *Daphne giraldii* Nitsche. Chin. Tradit. Herb. Drugs 39, 1781–1783.

Sun, Q., Li, F.F., Wang, D., Wu, J., Yao, G.D., Li, X., Li, L.Z., Liu, Q.B., Huang, X.X., Song, S.J., 2016. Flavans with cytotoxic activity from the stem and root bark of *Daphne giraldii*. RSC Adv. 6, 55919–55929. <https://doi.org/10.1039/C6RA08537G.>

Sun, Q., Yao, G.D., Song, X.Y., Qi, X.L., Xi, Y.F., Li, L.Z., Huang, X.X., Song, S.J., 2017. Autophagy antagonizes apoptosis induced by flavan enantiomers from *Daphne giraldii* in hepatic carcinoma cells in vitro. Eur. J. Med. Chem. 133, 1–10. https://doi.org/10.1016/j.ejmech.2017.03.072.

Zhao, J., Jin, X.J., Zhang, H.J., 2012. Research progress of *Daphne giraldii*. Chinese Wild Plant Resources 31, 12–14. https://doi.org/10.3969/j.issn.1006-9690- 2012.06.003.

Su, J., Zhang, C., Zhang, W., Shen, Y.H., Li, H.L., Liu, R.H., Zhang, X., Hu, X.J., Zhang, W.D., 2009. Qualitative and quantitative determination of the major coumarins in Zushima by high performance liquid chromatography with diode array detector and mass spectrometry. J. Chromatogr. A 1216, 2111–2117. https://doi.org/10.1016/j.chroma.2008.06.015.
